# Characterization of bacterial endophytes isolated from *Cannabis sativa* L. and *Chelidonium majus* L. for their application as biostimulants and biocontrol agents

**DOI:** 10.3389/frmbi.2026.1780965

**Published:** 2026-06-08

**Authors:** Mohammad Jamil Kaddoura, Laura Amaya-Quiroz, Mamta Rani, Zarna Shah, Kavya Reghunadh, Jamil Samsatly, Hacene Meglouli, Saji George

**Affiliations:** 1 Department of Food Science and Agricultural Chemistry, McGill University, Montreal, QC, Canada; 2 Biosun Solutions, Chambly, QC, Canada

**Keywords:** abiotic stress tolerance, *Bacillus*, bacterial endophytes, biological control, *Cannabis sativa*, *Chelidonium majus*, plant biostimulant, pseudomonas

## Abstract

Endophytic bacteria contribute to plant growth, stress tolerance, and pathogen resistance. Their effective use in agriculture requires the identification of strains that combine multiple beneficial traits with consistent performance across different field conditions. Accordingly, this study examines *Bacillus* and *Pseudomonas* endophytes isolated from *Cannabis sativa* L. and *Chelidonium majus* L. for plant growth promotion, abiotic stress tolerance, and biocontrol properties. Plant growth-promotion traits included indole, siderophore, and organic acid production, phosphate and zinc solubilization, and biofilm formation. Results showed that all the tested bacterial isolates produced indoles, with the highest levels recorded in *Pseudomonas* strain PPW-26, whereas several *Pseudomonas* strains exhibited strong siderophore production. Strain PPW-26 tested positive for methyl-red, indicating organic acid production, whereas other *Pseudomonas* strains tested negative. Moderate to high nutrient solubilization profiles were observed across all *Pseudomonas* strains. *Bacillus* strains, particularly BS-114, exhibited higher biofilm formation relative to *Pseudomonas*. Assessment of abiotic stress tolerance included proline accumulation, superoxide dismutase activity, and growth under varying temperature, salinity, and drought conditions. All strains displayed tolerance to the tested stresses, with *Bacillus* strains showing stronger resilience to high temperature and salinity, accompanied by elevated proline accumulation and superoxide dismutase activity in selected strains. Biocontrol potential was evaluated through biosurfactant production and antifungal activity. *Bacillus* strains showed high biosurfactant activity and strong inhibition of fungal pathogens. Strain BS-120 exhibited broad-spectrum inhibition against *Fusarium oxysporum*, *Fusarium graminearum*, and *Rhizoctonia solani*-AG3. Analysis of genome sequences identified biosynthetic gene clusters encoding antifungal metabolites, including fengycin and surfactin, consistent with the observed inhibition. Genome-wide similarity analysis and ANI-based clustering revealed the presence of highly similar and genetically distant strains within each genus. For *Bacillus* spp., ANI values ranged from 87.62% to 98.83%, whereas for *Pseudomonas* spp. they ranged between 83.91% and 99.99%, confirming the presence of substantial intra-genus diversity. Phylogenetic analysis showed well-supported clades consistent with ANI clustering. Overall, this study demonstrates that endophytic *Bacillus* and *Pseudomonas* strains exhibit complementary and strain-dependent traits associated with plant growth promotion, stress tolerance, and pathogen suppression, supporting their further evaluation as potential bioinoculants for sustainable agriculture.

## 1 Introduction

Endophytes are typically non-pathogenic microorganisms mainly bacteria, fungi, and archaea that exist within plants (Negi et al., 2024). These microorganisms exhibit a broad host range and are reported to be universally present in all plant species studied to date (Strobel and Daisy, 2003). Endophytes can reside in the seeds, flowers, fruits, leaves, stems, and roots of host plants (Compant et al., 2011; Qin et al., 2018). Bacterial endophytes can be considered a subgroup of rhizobacteria that have acquired the ability to colonize their hosts without causing any harm or disease symptoms. They can establish a symbiotic relationship with their host and support crop growth, health, and tolerance to biotic and abiotic stresses (Reinhold-Hurek and Hurek, 1998). Endophytes exert their beneficial effects through direct mechanisms, such as the production of growth regulators, nutrient solubilization, and enhanced nutrient availability, as well as indirectly through stimulation of systemic immune responses against pathogens (Lodewyckx et al., 2002). Diverse bioactive compounds produced by endophytes are increasingly recognized as valuable tools in sustainable agriculture (Sena et al., 2024).

Several studies have demonstrated that medicinal plants often harbor endophytes possessing similar activities, suggesting a correlation between the plant’s bioactive profile and its associated endophytic communities (Egamberdieva et al., 2017; Zhang et al., 2022; Khadka et al., 2024). In this context, medicinal plants with diverse secondary metabolites represent relevant systems for exploring functionally active endophytes. *Cannabis sativa* (cannabis) plants possess a diverse secondary metabolite profile, including terpenes and stilbenes, which have been associated with antimicrobial properties, and cannabinoids linked to therapeutic applications (Carvalho et al., 2023; O’Croinin et al., 2023; Berardo et al., 2024). Additionally, cannabis plants have been reported to host several bacterial endophytes, belonging to *Acinetobacter*, *Chryseobacterium*, *Enterobacter*, *Microbacterium*, and *Pseudomonas* genera (Gautam et al., 2013; Qadri et al., 2013; Afzal et al., 2015). Despite its potential scientific relevance, previous legal constraints in Canada and several U.S. states have hindered much of cannabis research. Current studies are primarily focused on topics such as medicine and pharmaceuticals, genetics, processing, policy, and management, whereas plant-microbiome interactions using omics-driven approaches account for less than 1% of published research (Vujanovic et al., 2020). Additionally, *Chelidonium majus* (greater celandine) is another medicinal plant of interest. It is found in Europe, Asia, and Northern Africa and possesses several bioactive compounds with pharmacological properties, such as anti-inflammatory, antimicrobial, immunomodulatory, anticancer, hepatoprotective, and analgesic effects (Gilca et al., 2010; Maji and Pratim Banerji, 2015). Given its reported activities, the plant’s chemical profile may contribute to structuring its associated microbial communities, potentially favoring endophytes with adaptive or functionally relevant traits. Similar to cannabis, studies on its endophytic communities and plant interactions remain limited, with a few reports identifying *Bacillus* spp. endophytes exhibiting biosurfactant and antifungal activity (Marchut-Mikolajczyk et al., 2018). These limited studies in both plants present several opportunities for further investigation.

The primary goal of this study was to identify and characterize bacterial endophytes isolated from *Cannabis sativa* and *Chelidonium majus*. Initial taxonomic identification was performed using 16S rRNA gene sequencing. The selected isolates were subsequently evaluated through a combination of phenotypic and genotypic approaches, including (i) plant growth-promoting traits, (ii) abiotic stress tolerance, (iii) biocontrol activity, and (iv) whole genome–based analyses. The identification of beneficial traits related to plant growth and protection in several of the studied isolates supports their potential application in plant agriculture as biostimulants and biocontrol agents.

## 2 Materials and methods

### 2.1 Plant material

Seeds of *Chelidonium majus* were sourced from Quebec, Canada. Stems and roots of 12-week-old *Cannabis sativa* strains *Baba-G* and *Candyland* were provided by a commercial grower in Quebec, Canada.

### 2.2 Isolation and purification of endophytic bacteria

All plant materials were surface sterilized by immersion in hydrogen peroxide (30% w/w) under agitation for 7 min, followed by several rinses in sterile distilled water (SDW). Sterilization success was verified using the imprint method, in which surface-sterilized tissues were pressed on Luria-Bertani (LB) agar and monitored for microbial growth. Several isolation methods were used to maximize the recovery of culturable endophytic bacteria, as detailed below.

*C. majus* seeds (100 mg) were sectioned using a sterile blade and incubated in 1 mL buffer solution (10–^1^ M, KH_2_PO_4_ + Na_2_HPO_4_) under agitation at 25˚C for 20 min, followed by serial dilution (10–^1^ to 10^-3^). Seed homogenates were prepared by grinding 300 mg in 3 mL of the same buffer and incubated under the same conditions, and serially diluted (10–^1^ to 10^-8^). All suspensions were plated on mannitol yolk polymyxin agar and nutrient agar supplemented with 1% sucrose. Similarly, *C. sativa* stems and root sections were incubated in buffer under agitation at 25˚C for 20 min, followed by serial dilution (10–^1^ to 10^-3^). Root homogenates were prepared by grinding 150 mg in 4 mL buffer and incubated under identical conditions, and serially diluted (10–^1^ to 10^-8^). All suspensions were plated on nutrient agar with 1% sucrose. All media plates were incubated at 25˚C for one week. Emerging bacterial colonies were purified through four successive rounds of single-colony isolation on LB agar amended with 10 mg.L^-1^ of the anti-fungal agent Benomyl (Wilson, USA). Pure isolates were stored in 25% (v/v) glycerol at -80 °C until further use.

The isolation and taxonomic identification of the endophytic bacterial strains described here, were previously reported (Amaya-Quiroz et al., 2026).

### 2.3 Molecular identification of bacterial endophytes (16S rRNA sequencing)

Bacterial strains were grown in LB to reach adequate cell concentrations. Genomic DNA was extracted from pelleted cells using the DNeasy Blood and Tissue Kit (Qiagen, Hilden, Germany), following the manufacturer’s instructions. The 16S rRNA gene sequences were amplified using the universal bacterial primers 27F (5′-AGAGTTTGATCCTGGCTCAG-3′) and 534R (5′-ATTACCGCGGCTGCTGG-3′) as previously described (Gagne‐Bourgue et al., 2013). PCR products were sequenced at Genome Quebec sequencing services (Montreal, QC, Canada) and queried against the NCBI database using BLASTN software to putatively identify 71 bacterial strains. Following 16S identification, seven *Bacillus* and six *Pseudomonas* strains were selected for subsequent screening.

### 2.4 Evaluation of plant-growth promoting traits in selected strains

#### 2.4.1 Total indole production

Total indoles were measured as described by Mohite (2013). For this, bacteria were cultured in LB with 0.1% tryptophan for 4 days. Culture supernatants were mixed with Salkowski reagent, incubated in dark for 2h, and absorbance was measured at 530 nm. Total indole content in the supernatant was quantified using an indole-3-acetic acid (IAA) standard calibration curve. The standard curve was generated by serial dilution of an IAA standard (Sigma-Aldrich, MO, USA) over a concentration range of 5-100 µg/mL (R² = 0.99).

#### 2.4.2 Siderophore production assay

Siderophore production was assessed using the chrome azurol S (CAS) agar assay in petri plates. Following spot inoculation with overnight cultures, the plates were incubated at 30 °C for 48 h, and yellow/brown halo diameters, indicative of siderophore activity, were measured at 24 and 48 h (Louden et al., 2011).

#### 2.4.3 Organic acid production assay

Organic acid production was measured using methyl red (MR) test and Voges-Proskauer (VP) tests, as previously described (McDevitt, 2009). Briefly, bacteria were cultured in glucose-phosphate broth for 4 days. A color change with MR indicated a positive result for acid production, while change following VP reagents (Barritt’s reagent A, 5% w/v α-naphthol in absolute ethanol; Barritt’s reagent B, 40% w/v KOH in deionized water) indicated a positive result for the presence of acetoin, an intermediate product of sugar fermentation.

#### 2.4.4 Phosphate solubilization assay

Phosphate solubilization was tested on general purpose agar supplemented with inorganic phosphate Ca_3_(PO_4_)_2_, adjusted to pH 7.2, as described by Verma, Ladha (Verma et al., 2001). Following spot inoculation with bacterial cells obtained from overnight cultures and incubation at 30 °C for 48 h, a clearance zone >1 mm around the bacterial colonies and/or yellowing of the media indicated positive solubilization.

#### 2.4.5 Zinc solubilization assay

Inorganic zinc solubilization was assessed using mineral salt medium supplemented with ZnO and ZnCO_3_, adjusted to pH 7.2, based on the method described by Yasmin, Hussain (Yasmin et al., 2021). Following spot inoculation with bacterial cells and incubation at 30 °C for 5 days, clearance zones indicated positive solubilization.

#### 2.4.6 Biofilm formation assay

Biofilm formation and quantification were performed as described with modifications (O'Toole, 2010). Briefly, 100 µL of overnight cultures were diluted (1:100) in LB, added to a 96-well tissue culture plate (Fisher Scientific, MA, USA) and incubated at 30 °C for 24 h. The suspensions were removed and the plate was rinsed with SDW to remove unattached cells. Fixation was performed using 100 µL of methanol for 15 min and biofilm staining was done with 125 µL of 0.1% crystal violet solution for 15 min followed by washing with SDW. For quantification, 125 μL of 30% acetic acid was added to solubilize the dye, incubated for 15 min, and then transferred to a new plate. Absorbance was measured at 550 nm using a Synergy HT plate reader (Bio-TEK, VT, USA), with 30% acetic acid used to account for background noise.

### 2.5 Evaluation of abiotic stress tolerance traits in selected strains

#### 2.5.1 Bacterial lysis

Bacterial lysis was performed according to a previously described method with modifications (Durán et al., 2015). Briefly, cell pellets were obtained following centrifugation (4000 rpm, 5 min) using 3-16PK centrifuge (Sigma-Aldrich, MO, USA) of overnight cultures and washing with 0.85% NaCl solution. The pellets were resuspended in lysis buffer (100 mM EDTA, 50 mM NaCl, pH 6.9) containing 1 mg.mL^-1^ lysozyme (Sigma-Aldrich, MO, USA), incubated for 30 min at 37 °C, followed by sequential centrifugations (5000 rpm, 10 min; 13,000 rpm, 15 min) to yield clarified lysates. Resulting lysates were used for subsequent intracellular metabolite analyses.

#### 2.5.2 Proline production assay

Proline quantification was carried out with a modified ninhydrin assay. Briefly, 1 mL bacterial lysate was mixed with 2 mL 1.25% ninhydrin (Sigma-Aldrich, MO,USA) in glacial acetic acid at 100 °C for 30 min (Shabnam et al., 2016). Absorbance was measured at 508 nm and values were quantified using a proline standard calibration curve according to manufacture’s instruction. A standard curve was generated by serial dilution of a proline standard (Sigma-Aldrich, MO, USA) over a concentration range of 0.06-0.5 mM (R² = 0.99).

#### 2.5.3 Superoxide dismutase production assay

Superoxide dismutase (SOD) activity was quantified by measuring the inhibition of nitroblue tetrazolium’s photochemical reduction (Donahue et al., 1997; Zahir et al., 2021). In a 96-well assay, 5 µL of bacterial lysate were mixed with 250 µL of reaction buffer (75 μM nitroblue tetrazolium, 20 μM riboflavin, 100 μM EDTA-Na_2_, 130 mM methionine). Light-dependent reactions were initiated by riboflavin addition under a 15-W fluorescent lamp, halted after 10 min by light removal. Absorbance at 560 nm determined nitroblue tetrazolium reduction, with one enzyme unit defined as 50% inhibition under standardized conditions. Controls included light-exposed and dark reactions without enzyme. The experiment was performed with four replicates per treatment.

#### 2.5.4 Bacterial growth under temperature, salinity, and drought stress

Bacterial growth was monitored after a 24 h incubation on LB agar under different conditions: temperature (30-60 °C), salinity (1-20% NaCl), and drought (6-20% polyethylene glycol), to assess tolerance to abiotic stress. Bacterial viability was quantified as described with minor modifications (Majumder et al., 2022). Briefly, 100 μL of overnight bacterial cultures were dispensed into each well of a 96-well plate, followed by 20 μL resazurin solution (0.15 mg.mL^-1^ PBS). Plates were incubated at 37 °C for 2 h after which fluorescence intensity (550 nm excitation and 590 nm emission) was measured using a Synergy HT plate reader (Bio-TEK, VT, USA). Growth was expressed as relative fluorescent units compared to normal growth conditions as the baseline. The experiment was performed with six replicates per treatment.

### 2.6 Evaluation of biocontrol properties in selected strains

#### 2.6.1 Biosurfactant production assay

Biosurfactant production was assessed using a modified oil displacement assay (Morikawa et al., 2000). Bacteria were cultured in 150 mL LB broth at 30 °C (180 rpm, 5 days). The supernatant was collected by centrifugation (8000 rpm, 10 min) then acidified to pH 2, and left overnight at 4 °C to precipitate lipopeptides. Pellets were collected by centrifugation and were freeze-dried in a FreeZone 18 Liter Freeze Dry System (LabConco, MO, USA). Dry pellets (100 mg) were resuspended in 70% methanol, and 10 μL of the resulting methanolic suspension was dispensed at the center of a Petri plate (100 x 15 mm) containing 30 mL of SDW topped with 10 μL of crude oil to form a thin layer. The oil displacement area, indicating surfactant activity, was measured using ImageJ software (National Institutes of Health, 2012). Triton X-100 (10 mg.mL^-1^) served as the positive control, with methanol and uninoculated LB medium as negative controls.

#### 2.6.2 Fungal confrontation assays

Inhibition of the selected pathogenic fungi (*Fusarium oxysporum*, *Fusarium graminearum*, and *Rhizoctonia solani*-AG3), was assessed using a confrontation assay with modifications (Gagne‐Bourgue et al., 2013). For this, potato dextrose agar plates were inoculated in the center with a 5 mm diameter mycelial plug taken from the edge of an actively growing fungal colony. A 5 mL volume of each bacterial culture (10^8^ CFU.mL^-1^) was deposited 25 mm from the fungal plug. Radial growth inhibition of the fungus was measured 5 days post inoculation and was calculated using the growth reduction equation,


$$\text{Inhibition}=\frac{\left(C-T\right)}{C}\times \;100$$

where C represents the control radial growth and T represents the radial growth in the presence of bacterial treatment.

### 2.7 Comparative genomic analysis and phylogenomics

Identification of the 13 endophytic bacterial strains by whole genome sequencing using Oxford Nanopore’s PromethION-24 platform was previously reported (Amaya-Quiroz et al., 2026). Genome assemblies were deposited under the accession numbers: *P. fluorescens* group sp. strain PF-1 (GCA_054481335.1), *P. wadenswilerensis* strain PPW-26 (GCA_054481295.1), *P. mohnii* strain PFM-34 (GCA_054481315.1), *P. mohnii* strain PFM-48 (GCA_054481275.1), *P. fluorescens* group sp. strain PF-69 (GCA_054481235.1), *P. sichuanensis* PS-72 (GCA_054481255.1), *B. subtilis* strain BS-114 (GCA_054481215.1), *B. subtilis strain* BS-115 (GCA_054481195.1), *B. subtilis* strain BS-116 (GCA_054481175.1), *B. subtilis* strain BS-118 (GCA_054481155.1), *B. mojavensis* strain BSM-119 (GCA_054481135.1), *B. subtilis* strain BS-120 (GCA_054481115.1), *B. subtilis* strain BS-121 (GCA_054481095.1).

Comparative genome libraries were constructed using NCBI BLAST+ to select 299 *Bacillus* and 141 *Pseudomonas* genomes. Pairwise genomic similarity was assessed using average nucleotide identity (ANI) calculated with the pyANI Python package and the ANIm method (MUMmer). The resulting ANI matrices were converted to distance matrices using SciPy in Python, and hierarchical clustering was applied to generate dendrograms representing genomic relatedness. Data visualization was performed in Python using Biopython, Matplotlib, SciPy, and Plotly in Google Colab to generate heatmaps and dendrograms illustrating pairwise similarity patterns and clustering relationships.

Multiple sequence alignments were performed using MAFFT (command-line interface). Maximum likelihood phylogenetic trees were constructed using IQ-TREE (v3.0.1) with 1000 bootstrap replicates, followed by visualization and annotation in iTOL (v7.0). *Bacillus subtilis* strain 168 and *Pseudomonas fluorescens* strain ATCC 13525 were used as reference strains for their respective genera. A representative subset of 41 *Bacillus* and 41 *Pseudomonas* strains was selected to capture genus-level diversity, while minimizing redundancy. These genomes were subjected to the same analytical pipeline, providing a reduced dataset that captures taxonomic structure and strain relationships.

Functional annotation and classification were performed using the COG and KEGG databases to predict metabolic pathways and orthologous gene clusters. Anti-SMASH (v7.0) was used to identify secondary metabolite biosynthetic gene clusters (BGCs). Additional genome annotations, functional assignments, and biological subsystem insights were investigated via RAST, while in-silico strain typing was performed via the MLST database.

### 2.8 Statistical analysis

Data processing and analyses were performed using Microsoft Excel and GraphPad Prism (v10.6.1) (GraphPad Software, CA, USA). One-way analysis of variance (ANOVA) was performed to assess significant differences among treatment means, followed by Tukey’s *post-hoc* test for pairwise comparisons (P< 0.05). Experiments were conducted with a minimum of three replicates unless otherwise specified. Details of statistical analyses, replicate numbers, and data presentation are provided in the corresponding figure captions.

## 3 Results

A total of 71 bacterial isolates were recovered from the tested plant tissues. Following 16S rRNA identification, seven isolates belonging to the genus *Bacillus* and six to *Pseudomonas* were selected based on taxonomic classification for further characterization.

### 3.1 Evaluation of plant-growth promoting traits in selected strains

**Figure 1:**
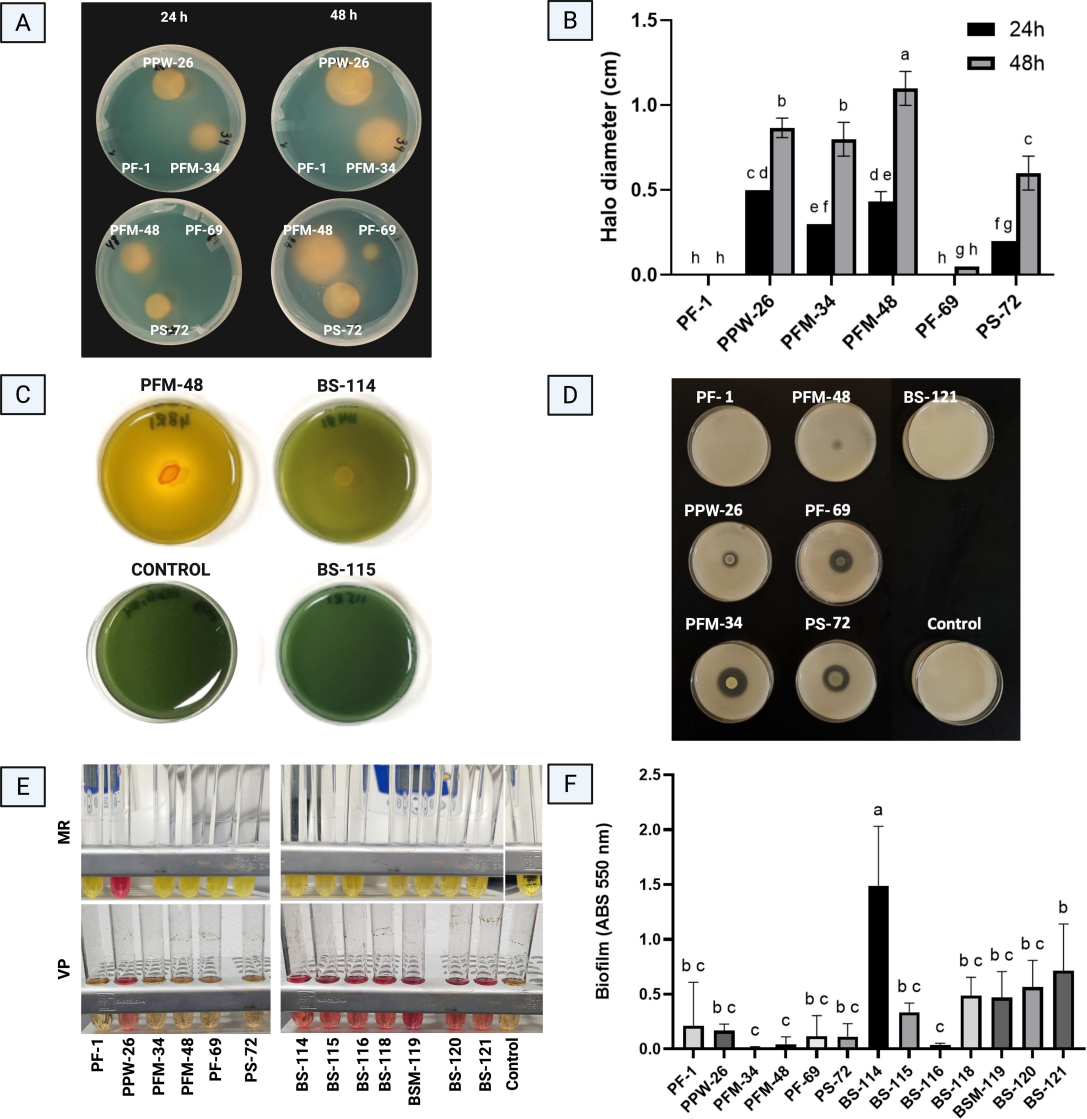
**(A)** Siderophore activity on CAS medium. **(B)** Halo diameter (cm) on CAS medium at 24 h and 48 h. **(C)** Phosphate solubilization on PDA with 0.75 g/L BTB. **(D)** Zn solubilization **(E)** Organic acid production in MR (top) and VP (bottom) assays. **(F)** Bacterial biofilm production after 24 h. Values represent mean ± SD (n= 3). Different letters indicate significant difference among treatments according to one-way ANOVA (Tukey’s HSD test, P < 0.05).

The seven *Bacillus* and six *Pseudomonas* strains displayed varying performance in the tested plant-growth promotion assays. All *Pseudomonas* strains exhibited moderate total indole production, with PPW-26 showing the highest levels (110.0 µg mL^-1^) and PS-72 the lowest (3.27 µg mL^-1^), whereas all *Bacillus* strains showed low indole production (7.0-12.0 µg mL^-1^), except BS-114, which exhibited moderate production (55.0 µg mL^-1^) (**Table 1**). For siderophore production, *Pseudomonas* strain PFM-48 showed the highest activity, with a visible yellow zone around the inoculation area at 24 h post-inoculation that further expanded after 48 h. PPW-26, PFM-34, and PS-72 exhibited moderate production, while no siderophore production was detected in any *Bacillus* strains (**Table 1**, **Figures 1A, B**). Regarding organic acid production, only PPW-26 tested positive for both MR and VP tests, as indicated by a color change from yellow to red. All *Bacillus* strains tested positive for the VP test, whereas the remaining *Pseudomonas* strains tested negative (**Table 1**, **Figure 1E**). For Phosphate solubilization, all *Pseudomonas* strains exhibited high activity, as indicated by a color change of the medium from green/blue to yellow and the presence of a prominent measurable halo around the colonies. Low activity was observed in BS-114, while no activity was detected in the remaining *Bacillus* strains (**Table 1**, **Figure 1C**). Similarly, for zinc solubilization, *Pseudomonas* strains PFM-34, PF-69, and PS-72 exhibited the highest activity, whereas PFM-48 showed low activity levels. No activity was detected in any *Bacillus* strains (**Table 1**, **Figure 1D**). All strains were identified as ‘positive’ for biofilm formation. *Bacillus* strains produced significantly more biofilm at 24 h than *Pseudomonas* strains, and BS-114 was the highest biofilm producer among the evaluated strains (**Figure 1F**).

**Table 1:** Summary of the production of total indoles, siderophores, and organic acids, along with the phosphate and zinc solubilization abilities of the bacterial strains.

Strain	Total Indole Production	Siderophores Production	Organic Acid Production	Phosphate Solubilization	Zinc Solubilization
PF-1	++	–	-/-	++	–
PPW-26	+++	++	+/+	++	++
PFM-34	++	++	-/-	++	+++
PFM-48	++	+++	-/-	++	+
PF-69	++	+	-/-	++	+++
PS-72	+	++	-/-	++	+++
BS-114	++	–	-/+	+	–
BS-115	+	–	-/+	–	–
BS-116	+	–	-/+	–	–
BS-118	+	–	-/+	–	–
BSM-119	+	–	-/+	–	–
BS-120	+	–	-/+	–	–
BS-121	+	–	-/+	–	–

Levels of production/solubilization were assigned based on assay-specific criteria. Indole production levels were categorized based on IAA-equivalent concentrations as follows: + (low,<15 µg mL^-1^), ++ (moderate, 15–<60 µg mL^-1^), and +++ (high, ≥60 µg mL^-1^). Siderophore production at 48 h were categorized based on clearance zone diameter as follows: − (no activity), + (low,<0.1 cm), ++ (moderate, 0.1–<1.0 cm), and +++ (high, ≥1.0 cm). Organic acid was assessed using methyl red (MR) and Voges Proskauer (VP) assays. Color change indicated a positive result (+). Data represented results for MR and VP respectively. Phosphate solubilization levels were categorized as follows: − (no activity), + (low activity = color change), ++ (high activity = color change & clearance zone > 1 mm). Zinc solubilization levels were categorized based on clearance zone diameter as follows: − (no activity), + (low,<1.0 cm), ++ (moderate, 1–<1.5 cm), and +++ (high, ≥1.5 cm).

### 3.2 Evaluation for abiotic stress tolerance traits in selected strains

**Figure 2:**
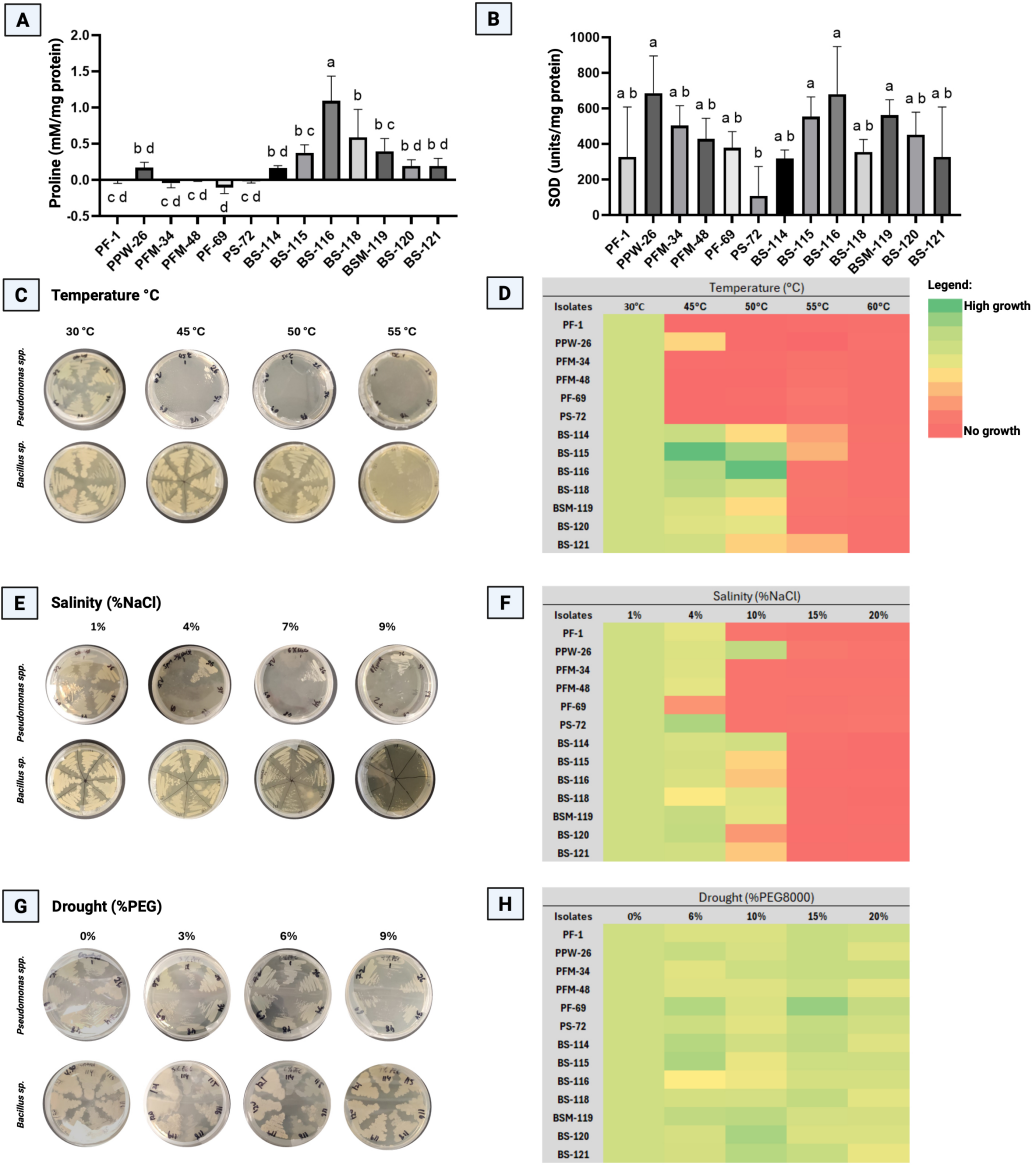
**(A)** Proline content (mM.mg-1 protein) in bacterial strains grown in LB media. **(B)** SOD activity (units.mg-1 protein) in bacterial strains. Bacterial growth on LBA plates under different **(C)** temperatures; **(E)** salinity; **(G)** and drought levels. Heat map summarizing bacterial growth under **(D)** 4, 30, 45, 50, 55 and 60 °C; **(F)** 1, 4, 7 and 9% NaCl; and **(H)** 6, 10, 15 and 20% polyethylene glycol. Values represent mean ± SD (A, B, n= 4; E, n= 6). Different letters indicate significant difference among treatments according to one-way ANOVA (Tukey’s HSD test, P < 0.05).

Under normal conditions, intracellular proline levels varied across strains, with accumulation detected in PPW-26 and all *Bacillus* strains, while no accumulation was observed in the other *Pseudomonas* strains (**Figure 2A**). The highest value was recorded in BS-116 (1.10 mM.mg^-1^ protein), followed by BS-118 (0.59 mM.mg^-1^ protein), while lower levels (0.16-0.40 mM.mg^-1^ protein) were observed in the remaining *Bacillus* strains *and Pseudomonas* strain PPW-26. Similarly, all strains exhibited SOD activity with the highest levels detected in PPW-26 and BS-116 (**Figure 2B**). Bacterial growth on LBA plates varied under different temperature, salinity, and drought conditions (**Figures 2C, E, G**). Thermotolerance differed among genera, with only *Pseudomonas* strain PPW-26 growing at 45 °C, while all *Bacillus* strains grew at 50 °C (**Figures 2C, D**). BS-114, BS-115, and BS-121 demonstrated the most notable thermotolerance, retaining measurable growth at 55 °C. Less variation was observed in salinity tolerance, as all *Pseudomonas* and *Bacillus* strains were viable at 4% NaCl, except PF-69, which showed limited growth at this concentration. PPW-26, BS-114, BS-118, and BSM-119 displayed higher salinity tolerance and grew at 10% NaCl (**Figures 2E, F**). Polyethylene glycol, an osmotically active compound that lowers water potential and limits water availability, was used to simulate drought conditions. All bacterial strains exhibited measurable growth under polyethylene glycol-induced drought conditions at the tested levels in LB (**Figures 2G, H**). Overall, bacterial strains BS-114, BS-115, BS-118, BSM-119, BS-121, and PPW-26 showed the highest viability under the tested abiotic stress conditions including high temperatures, high salinity, and drought.

### 3.3 Evaluation of biocontrol properties in selected strains

**Figure 3:**
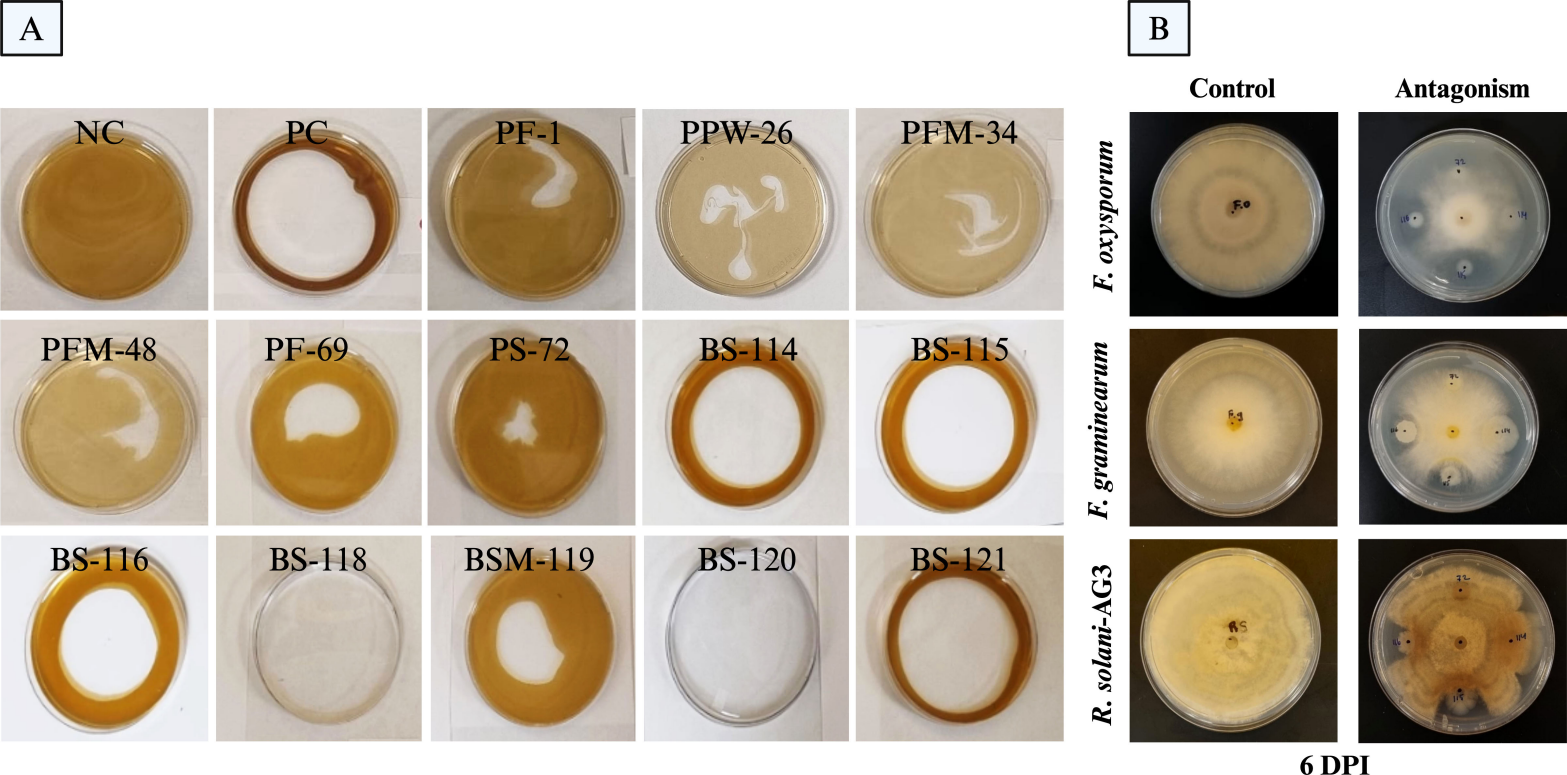
**(A)** Oil dispersal activity of tested *Pseudomonas* strains (PF-1, PPW-26, PFM-34, PFM-48, PF-69, and PS-72); *Bacillus* strains (BS-114, BS-115, BS-116, BS-118, BSM-119, BS-120, BS-121); NC, negative control (media); and PC, positive control (Triton X). **(B)** Antagonistic activity of bacterial strains against the fungal pathogens *Fusarium oxysporum*, *Fusarium graminearum*, and *Rhizoctonia solani* (AG3) at 6 DPI.

All bacterial strains were identified as biosurfactant producers using the oil-spreading assay (**Table 2**, **Figure 3A**). Biosurfactant activity was categorized into different levels (high, medium, and low) based on the oil dispersal observed. *Bacillus* strains showed the highest biosurfactant activities, with most classified in medium (>25% and<70%) to high activity (≥70%) categories, while all *Pseudomonas* strains categorized as low (≤25%) in biosurfactant production. BS-120 and BS-118 exhibited the highest activity, comparable to the positive control, Triton X. This was followed by BS-121, BS-115, BS-114, and BS-116 which displayed medium activity.

**Table 2:** Biosurfactant production of bacterial strains.

Strain	% Average Oil Dispersal
PF-1	8.0 ± 0.1 (c)
PPW-26	21.5 ± 0.4 (c)
PFM-34	6.4 ± 0.3 (c)
PFM-48	10.1 ± 0.1 (c)
PF-69	16.6 ± 0.1 (c)
PS-72	4.4 ± 0.0 (c)
BS-114	55.7 ± 0.2 (b)
BS-115	55.8 ± 0.1 (b)
BS-116	43.3 ± 0.1 (b)
BS-118	93.1 ± 0.5 (b)
BSM-119	20.3 ± 0.2 (c)
BS-120	97.3 ± 0.3 (a)
BS-121	66.0 ± 0.1 (b)

For the oil spreading assay, dispersal was categorized as follows: (a), high activity (≥70%); (b), medium activity (>25% and<70%); and (c), low activity (≤25%). Values represent the mean of three replicates ± standard error of the mean (S.E.).

In the antifungal assays, the *Bacillus* strains exhibited the highest activity, with varying levels of inhibition observed (**Table 3**, **Figure 3B**). BS-120, BS-115, and BS-119 showed the highest inhibition against all fungal pathogens, including *F. oxysporum*, *F. graminearum*, and *R. solani-*AG3. Inhibition was indicated by a clear zone of growth suppression around the colony, which developed within 24–48 h and persisted throughout the experiment, suggesting the production of effective antimicrobial compounds. Additionally, BS-116 and BS-121 exhibited strong inhibition against all fungal isolates, except for *F. oxysporum* and *R. solani*, where they fell below the set threshold (≥40% inhibition). In contrast, the *Pseudomonas* strains showed no notable inhibition against any of the fungal species tested.

**Table 3:** Antagonistic activity of bacterial strains against the fungal pathogens, *Fusarium oxysporum*, *Fusarium graminearum*, and *Rhizoctonia solani* (AG3).

Strain	% Inhibition		
	*Fusarium oxysporum*	*Fusarium graminearum*	*Rizoctonia solani* – AG3
PF-1	9.2 ± 1.0	0.0 ± 0	11.7 ± 1.6
PPW-26	0.0 ± 0	0.0 ± 0	0.0 ± 0
PFM-34	0.0 ± 0	0.0 ± 0	0.0 ± 0
PFM-48	6.7 ± 0.5	0.0 ± 0	0.0 ± 0
PF-69	0.0 ± 0	0.0 ± 0	21.7 ± 2.6
PS-72	3.3 ± 0.7	0.0 ± 0	14.2 ± 2.0
BS-114	10.0 ± 1.1	52.3 ± 0.9 (a)	19.7 ± 1.7
BS-115	42.2 ± 0.3 (a)	53.4 ± 0.4 (a)	43.1 ± 1.3 (a)
BS-116	34.6 ± 0.1	52.7 ± 0.3 (a)	41.7 ± 1.7 (a)
BS-118	37.7 ± 0.3	41.5 ± 0.8 (a)	38.5 ± 2.1
BSM-119	48.8 ± 0.4 (a)	49.0 ± 1.5 (a)	43.2 ± 2.4 (a)
BS-120	55.5 ± 0.3 (a)	52.5 ± 1.3 (a)	45.2 ± 1.8 (a)
BS-121	58.0 ± 1.5 (a)	44.7 ± 0.9 (a)	38.3 ± 2.2

For the confrontation assay, fungal inhibition > 40% was considered positive and denoted as (a). Values represent the mean of three replicates ± standard error of the mean (S.E.).

### 3.4 Comparative genomic analysis and phylogenomics

**Figure 4:**
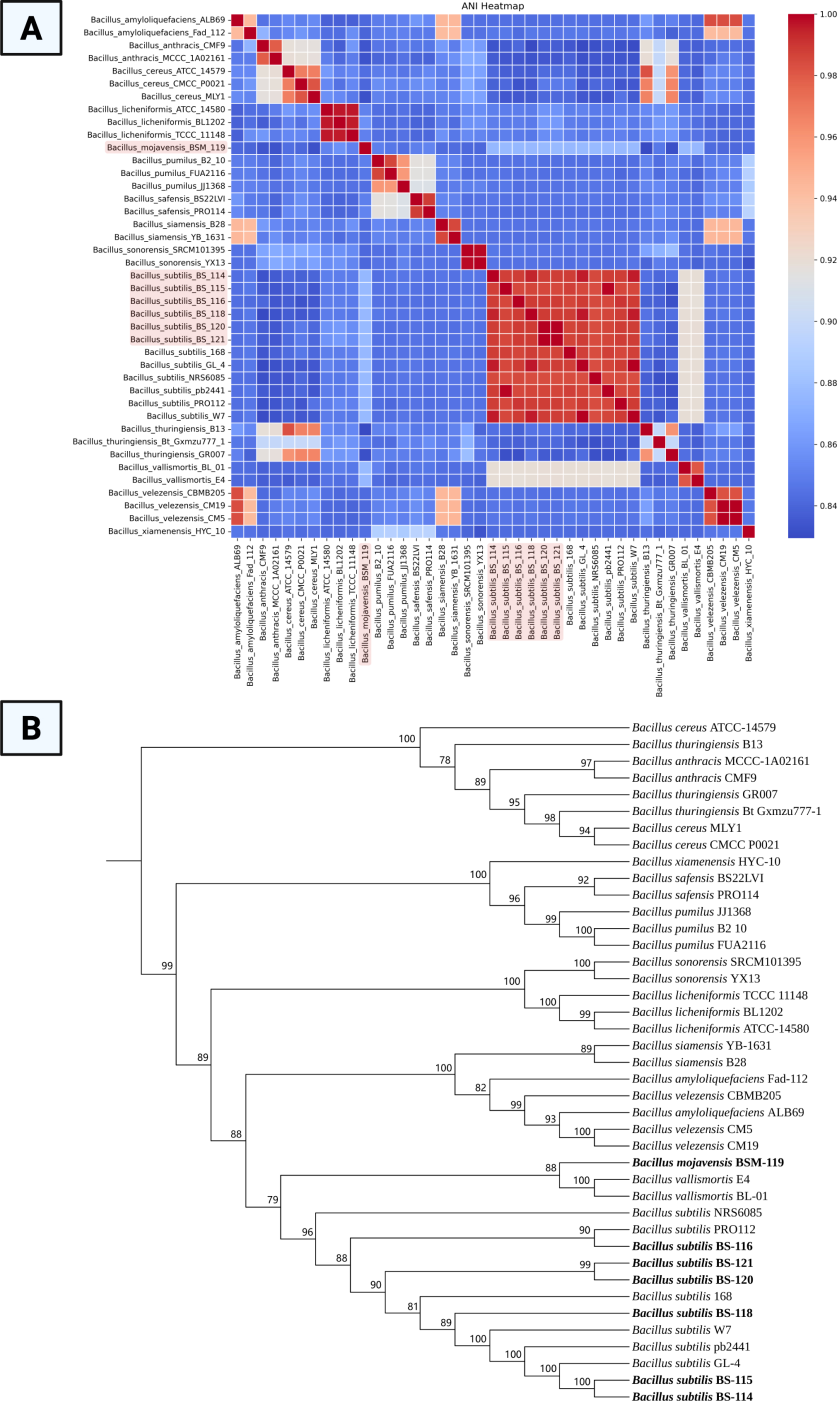
**(A)** ANI heatmap representing the similarity between the isolated strains of the study along with selected representative *Bacillus* strains from the database. Red and blue in the heatmap indicate high and low correlation, respectively. **(B)** Phylogenetic tree with bootstrap simulations comparing the relation and similarity of the study’s strains and the representative *Bacillus* strains.

**Figure 5:**
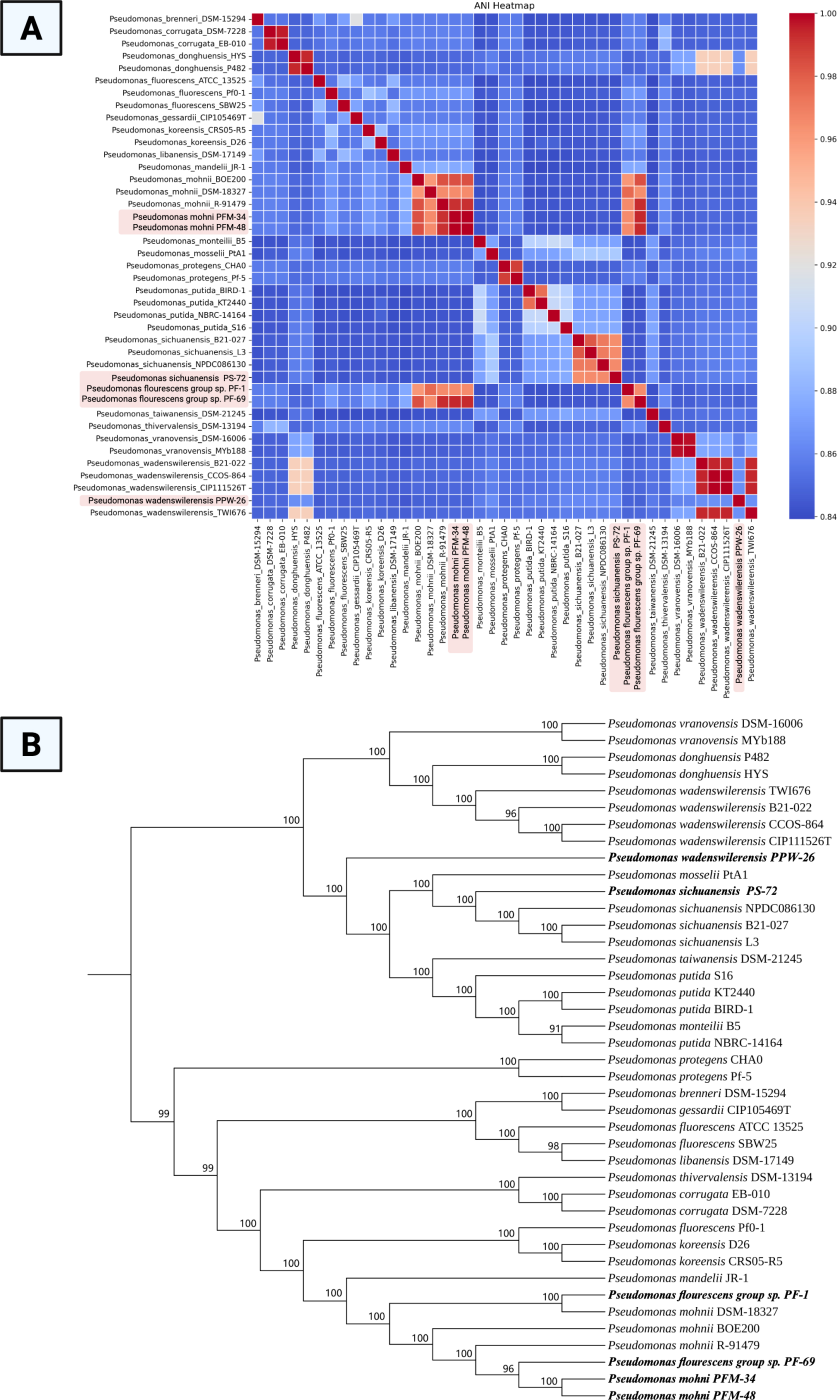
**(A)** ANI heatmap representing the similarity between the isolated strains of the study along with selected representative *Pseudomonas* strains from the database. Red and blue in the heatmap indicate high and low correlation, respectively. **(B)** Phylogenetic tree with bootstrap simulations comparing the relation and similarity of the study’s strains and the representative *Pseudomonas* strains.

Strains BS-114, BS-115, BS-116, BS-118, BS-120, and BS-121 exhibited high genomic similarity with ANI values >98.63% with *Bacillus subtilis*, confirming their classification within this species. Meanwhile, BSM-119 displayed an ANI of 87.63%, falling below the species demarcation threshold, suggesting it may represent a distinct taxonomic group. ANI values against other *Bacillus* species, including *B. vallismortis, B. licheniformis*, *B. cereus*, *B. pumilus*, *B. velezensis*, and *B. amyloliquefaciens*. remained below 91% for all strains, reinforcing their genetic divergence from these taxa (**Figure 4A**). A similar analysis was conducted for the *Pseudomonas* strains with none of the analyzed strains meeting the threshold for species-level identity (**Figure 5A**). Strain PPW-26 and PS-72 exhibited the highest ANI values with *P. sichuanensis*, 86.07% and 88.93% respectively, indicating a closer genetic relationship. Meanwhile, PF-1, PFM-34, PFM-48, and PF-69 showed higher genetic similarity to *P. jessenii* (>87.63%) and *P. mandelii* (>87.52%). Additionally, strain PPW-26 displayed the lowest ANI values with the reference strains suggesting the greatest genetic divergence. Hierarchical clustering of the ANI-derived distance matrices revealed several distinct clades. Heatmaps and dendrograms depicted clear clusters of strains sharing high genomic similarity, while other strains formed outlier groups, suggestive of potential novel lineages. These results highlight the considerable genomic diversity among BSM-119 and *Pseudomonas* strains, suggesting that further taxonomic resolution is needed to refine the classification of these strains.

Phylogenetic analyses further supported these findings by clustering most *Bacillus* strains within the main *B. subtilis* clade, closely related to the reference strain 168, while BSM-119 formed a distinct branch, highlighting its genomic uniqueness (**Figure 4B**). Similarly, *Pseudomonas* strains PFM-34, PFM-48, PF-69, and PF-1 clustered with *P. mohni* clade, and PS-72 grouped near *P. sichuanensis* clade, whereas PPW-26 occupied a divergent clade (**Figure 5B**). The observed taxonomic placement, combined with the identification of novel sequence types through MLST, underscores the considerable genomic diversity present among these isolates (**Table 4**). Collectively, these results suggest that while most of the identified *Bacillus* represent novel strains or variants within *B. subtilis*, BSM-119 and several *Pseudomonas* isolates, particularly PPW-26, may constitute novel subspecies or distinct taxonomic entities warranting further investigation.

**Table 4:** Taxonomic assignment, MLST status, and GenBank accession numbers of the 13 bacterial isolates.

Strain	Taxonomic ID	Genome Accession	MLST
PF-1	*Pseudomonas fluorescens* group sp.	JBTMNG000000000	Novel
PPW-26	*Pseudomonas wadenswilerensis*	JBTMNF000000000	Novel
PFM-34	*Pseudomonas mohnii*	JBTMNE000000000	Novel
PFM-48	*Pseudomonas mohnii*	JBTMND000000000	Novel
PF-69	*Pseudomonas fluorescens* group sp.	JBTMNC000000000	Novel
PS-72	*Pseudomonas sichuanensis*	JBTMNB000000000	Novel
BS-114	*Bacillus subtilis*	JBTMNA000000000	Novel
BS-115	*Bacillus subtilis*	JBTMMZ000000000	Novel
BS-116	*Bacillus subtilis*	JBTMMY000000000	Novel
BS-118	*Bacillus subtilis*	JBTMMX000000000	Novel
BSM-119	*Bacillus mojavensis*	JBTMMW000000000	Novel
BS-120	*Bacillus subtilis*	JBTMMV000000000	Novel
BS-121	*Bacillus subtilis*	JBTMMU000000000	Novel

Only genomic regions showing 100% sequence homology to known BGCs were reported, supporting the identification of relevant secondary metabolites (**Table 5**). Different gene clusters, including non-ribosomal peptide synthases and polyketide synthases, along with antimicrobial resistance profiles were observed. *Bacillus* strains BS-114, BS-115, BS-116, BS-118, BS-120, and BS-121 were predicted to harbor non-ribosomal peptide synthases, such as bacillibactin, fengycin, and surfactin and carried the resistance genes *aadK*, *mph(K)*, *tet(L)*. In contrast, the *Pseudomonas* strain PPW-26 was predicted to encode pseudomonine.

**Table 5:** Predicted biosynthetic gene clusters (BGCs) in isolated strains and predicted antimicrobial resistance genes.

Strain	NRPS	PKS	Others	AMR	Resistance
PF-1	n.d.	n.d.	n.d.	n.d.	n.d.
PPW-26	Pseudomonine	n.d.	n.d.	n.d.	n.d.
PFM-34	n.d.	n.d.	n.d.	n.d.	n.d.
PFM-48	n.d.	n.d.	n.d.	n.d.	n.d.
PF-69	n.d.	n.d.	n.d.	n.d.	n.d.
PS-72	n.d	n.d.	Hydrogen cyanide	n.d.	n.d.
BS-114	Bacillibactin, Fengycin, Pulcherriminic acid	Bacillaene	Subtilin, Subtilocin A, Bacilysin	aadK, mph(K), tet(L)	streptomycin, spiramycin, telithromycin, tetracycline, doxycycline
BS-115	Bacillibactin, Pulcherriminic acid Surfactin,	Bacillaene	Subtilomycin, Subtilocin A, Bacilysin	aadK, mph(K), tet(L)	streptomycin, spiramycin, telithromycin, tetracycline, doxycycline
BS-116	Bacillibactin, Fengycin	Bacillaene	Subtilocin A, Bacilysin	aadK, mph(K)	streptomycin, spiramycin, telithromycin
BS-118	Bacillibactin, Fengycin, Pulcherriminic acid	Bacillaene	Subtilin, Subtilocin A, Bacilysin	aadK, mph(K)	streptomycin, spiramycin, telithromycin
BSM-119	Bacillibactin, Fengycin, Pulcherriminic acid, Surfactin	n.d.	Subtilocin A, Bacilysin	n.d.	n.d.
BS-120	Bacillibactin, Fengycin, Pulcherriminic acid, Surfactin	Thailanstatin A	Subtilin, Subtilocin A, Bacilysin	aadK, mph(K)	streptomycin, spiramycin, telithromycin
BS-121	Bacillibactin, Fengycin, Pulcherriminic acid	n.d.	Subtilin, Subtilocin A, Bacilysin	aadK, mph(K)	streptomycin, spiramycin, telithromycin

NRPS, non-ribosomal peptide synthetase; PKS, polyketide synthase; AMR, antimicrobial resistance; n.d., not detected.

The integrative use of these bioinformatics tools provides genomic and predicted functional evidence supporting targeted investigation of strain-specific traits and biotechnological applications.

## 4 Discussion

The careful selection of beneficial microorganisms is essential for the development of successful microbial formulations for agriculture applications. Notably, effective bacterial strains often express characteristics that collectively contribute to their performance, such as growth and survival traits, nutrient mobilization and acquisition potential, and host compatibility (Vasseur-Coronado et al., 2021). Bacterial endophytes previously identified by whole-genome sequencing, isolated from *C. sativa* and *C. majus* were initially reported in a genome announcement (Amaya-Quiroz et al., 2026), which generated the WGS datasets used herein. The aim of this study was to further explore these datasets and the functional characterization of these endophytic strains for plant-beneficial properties, including growth promotion, resilience to abiotic stresses, and protection against pathogens. Several strains displayed characteristics that highlight their potential for crop improvement and protection.

### 4.1 Evaluation of plant-growth promoting traits in selected strains

Total indoles were assessed due to the widespread prevalence of indole-3-acetic acid among plant growth-promoting (PGP) bacteria and its roles in enhancing plant-bacterial interactions, root development, colonization, and nutrient availability (Etesami et al., 2015). Since nutrient presence in the soil does not necessarily translate to plant bioavailability, microbial traits that enhance nutrient acquisition are particularly valuable. Consequently, bacterial mechanisms such as soil pH modulation, solubilization of insoluble macronutrients (e.g., phosphorus) and micronutrients (e.g., zinc) and iron chelation represent key PGP traits (Zaheer et al., 2019; Sun et al., 2022). In addition to these PGP traits, characteristics related to bacterial persistence and plant root colonization, such as biofilm formation, were also evaluated.

All *Bacillus* and *Pseudomonas* strains evaluated in this study produced indoles, with levels ranging from low to moderate, and with higher levels observed in certain strains such as *Pseudomonas* PPW-26, consistent with previous reports for these genera (Khan et al., 2016; Meliani et al., 2017). Indole-3-acetic acid is among the most extensively reported mechanisms associated with PGP bacteria. It has been implicated in enhancing bacterial fitness by improving tolerance to environmental stresses, supporting biofilm formation, and root colonization (Pantoja-Guerra et al., 2023). From the host perspective, bacterial-derived IAA can locally modulate auxin signaling, leading to enhanced lateral root length and root hair formation, consequently improving nutrient acquisition. In addition, IAA-mediated signaling has been associated with increased chlorophyll content and modulation of plant defense responses, contributing to improved plant performance under both optimal and stress conditions (Feng et al., 2024).

Additionally, strains belonging to *P. mohni*, *P. putida*, and *P. sichuanensis* produced siderophores. No activity was observed in the *B. subtilis* strains in the CAS assays, despite WGS revealing the presence of bacillibactin BGCs and existing reports of catecholate siderophore production in this genus (Nithyapriya et al., 2021). In comparison, siderophore production by several *Pseudomonas* species, has been widely reported and linked to improved plant performance and increased tolerance to abiotic stress (Sandhya et al., 2009; Syed et al., 2023). Siderophore production in *Pseudomonas* is predominantly mediated by pyoverdine, a high-affinity iron chelator (Vindeirinho et al., 2021). *Pseudomonas* strains isolated from cannabis *have also been reported for siderophore production* (Scott et al., 2018). In support of these observations, genes associated with siderophore biosynthesis and modification (*entC*, *entD*, and *pchB*) were identified within KEGG pathway 01053 in these genomes (Crosa and Walsh, 2002).

*Pseudomonas* strain PPW-26 tested positive for organic acid production through MR test. Indicating an accumulation of exogenous acid production. Organic acid production (e.g., oxalic, gluconic, and formic acid) is commonly reported among PGP bacteria which can enhance the bioavailability of inorganic mineral forms, including phosphorus and zinc, through soil pH modulation (Wei et al., 2018). These effects occur through proton-mediated mineral dissolution, metal cation chelation, and altered nutrient speciation.

Several *Pseudomonas* strains displayed moderate to high phosphate solubilization activity, as evidenced by the formation of clearance zones and a change in the color of the medium. This is likely associated with organic acid production and release into the culture, resulting in localized pH reductions. In contrast, among the *Bacillus* strains, a single strain exhibited phosphate solubilization, and this activity was limited. Phosphate solubilization efficiency is influenced by multiple factors such as organic acid presence, type, and concentration, as well as additional solubilization mechanisms and enzymes (Pan and Cai, 2023). Environmental conditions, including temperature, have also been shown to influence phosphate solubilization, with variable effects reported in the literature (White et al., 1997; Nautiyal et al., 2000; Fasim et al., 2002). Moreover, the chemical form of insoluble phosphorus plays a critical role in assessment outcomes; therefore, employing a combination of phosphorus sources tailored to specific soil types can improve evaluation accuracy (Bashan et al., 2013). Notably, some bacterial strains have been reported to effectively solubilize and release phosphate in soil despite showing no halo zones in agar plate assays (Liu et al., 2015).

A similar trend was observed for zinc solubilization. Among the *Bacillus* strains, a single strain exhibited limited solubilization activity, whereas several *Pseudomonas* strains demonstrated greater solubilization capacity, ranging from low to moderate. These findings align with previous reports indicating that *Pseudomonas* species show higher solubilization efficiency in the presence of zinc oxide (ZnO) and zinc carbonate (ZnCO_3_), while *Bacillus* species have been reported to preferentially solubilize zinc sulfide (ZnS) (Saravanan et al., 2004).

Taken together, these findings highlight the multifactorial nature of nutrient solubilization, which is governed by interacting biological and environmental processes that may not be fully captured when examined in isolation. Consequently, conventional plate-based assays may not adequately reflect the nutrient-solubilizing potential of PGP bacteria under soil-relevant conditions.

Biofilms are microbial communities that adhere to surfaces and are embedded in self-produced extracellular polymeric substances, conferring advantages such as enhanced resilience, surface adhesion, and root colonization (Flemming and Wingender, 2010). Biofilm formation has been widely associated with increased tolerance to environmental stresses and contributes to pathogen control by enabling rapid and stable root colonization, thereby limiting pathogen establishment (Masmoudi et al., 2019; Di Francesco et al., 2021). In this study, *Bacillus* strains produced significantly more biofilm than *Pseudomonas*, consistent with previous research (Jain et al., 2013; Masmoudi et al., 2019). This difference may be attributed to the hydrophobic cell surface properties of *Bacillus*, which enhance adhesion and biofilm structural stability (Catania et al., 2023). KEGG annotation further identified genes associated with biofilm formation in *B. subtilis* within the 02024 quorum-sensing pathway, including *Spo0A*, a key regulator of sporulation and biofilm formation under stress or nutrient-limitation (Milton and Cavanagh, 2023), and *ComA*, a quorum-sensing regulator reported to control genes involved in biofilm formation and other collective bacterial behavior (Kalamara et al., 2018).

The examined strains exhibited a wide range of PGP traits that collectively support effective root colonization and persistence within plant tissues. Through these traits, they may promote plant growth via direct, indirect, or synergistic mechanisms, depending on environmental context and host plant interactions (Kaddoura et al., 2026).

### 4.2 Evaluation of abiotic stress tolerance traits in selected strains

While endophytes may exhibit beneficial traits, their ability to maintain metabolic activity and functional performance under adverse conditions is critical for their effectiveness as biostimulants. Assessing bacterial responses to abiotic stress thus provides insight into their resilience under agriculturally relevant conditions. Accordingly, the strains were evaluated for tolerance to high temperature, salinity, and drought by assessing proline accumulation, SOD activity, and growth rate under varying stress conditions.

Proline, a well-documented osmoprotectant, is essential for cellular stability under osmotic stress (Viscardi et al., 2016). In this study, *Bacillus* strains accumulated higher proline levels than *Pseudomonas*, consistent with previous reports of strong osmoregulatory capacity in this genus (Mahipant et al., 2017; Metoui Ben Mahmoud et al., 2020). Notably, strain PPW-26 also exhibited elevated proline levels, comparable to those observed in *Bacillus* strains. The accumulation of proline under non-stressed conditions suggests a preemptive strategy that may enhance tolerance to osmotic stress (Goswami et al., 2022; Navarro-Torre et al., 2023). At high concentrations, proline synthesis can be downregulated, and excess proline can be released, hence potentially contributing to plant utilization (Roy et al., 1993; Goswami et al., 2022). Beyond its osmoprotective role, proline contributes to stress tolerance by stabilizing proteins and membranes, chelating metals, functioning as a signaling molecule, and supporting antioxidative defense under drought and salinity stress (Ashry et al., 2022). The observed proline production is supported by genomic analysis, as key genes involved in biosynthesis were identified in KEGG pathway 00330 (arginine and proline metabolism), including *proA*, *proB*, and *proC*. In *B. subtilis*, proline synthesis is essential for osmotic adaptation and is mediated primarily via the glutamate-dependent pathway (Goswami et al., 2022; Stecker et al., 2022), whereas alternative biosynthetic routes, such as ornithine-dependent proline synthesis have been reported in *P. putida* (Jensen and Wendisch, 2013), which may contribute to the observed proline phenotype in these strains.

SOD activity is a key indicator of a bacteria’s ability to counteract oxidative damage caused by reactive oxygen species (Koza et al., 2022). In this study, all examined strains exhibited SOD activity, suggesting an enhanced capacity for survival under challenging conditions. Genomic analysis identified the *sodA* gene (K04564) in *B. subtilis* and *sodC* (K04565) in *Pseudomonas* strains, both annotated in KEGG under Environmental Information Processing category (FOXO signaling pathway), which encompasses cellular responses to oxidative stress. In *B. subtilis*, *sodA* is expressed in both vegetative cells and spores, where it contributes to oxidative stress tolerance, particularly during sporulation and germination (Cao et al., 2005; Inaoka et al., 2014).

Growth performance under abiotic stress provides a comprehensive measure of strain viability and adaptive capacity. Among the tested bacteria, several strains maintained measurable growth under elevated temperature, salinity, and drought-associated conditions, indicating functional resilience. Notably, *Bacillus* strains and the *Pseudomonas* strain PPW-26 exhibited superior growth across multiple stress conditions, highlighting their potential relevance to support plant performance across a range of challenging environmental conditions.

The observed growth resilience is supported by genomic features associated with stress adaptation rather than a single metabolite. Both *Bacillus* and *Pseudomonas* strains harbored genes involved in osmoregulation and synthesis of compatible solutes, including glycine betaine-related genes *gbsA* and *betB* (K00130), which support cellular stability and sustained growth under osmotic stress (Kempf and Bremer, 1998). In *Pseudomonas* strains, the presence of *gsh* gene (K01920) involved in glutathione biosynthesis, further supports oxidative stress mitigation during active growth. Additionally, both genera contain two-component regulatory systems such as *phoB*, that modulate gene expression in response to osmotic and thermal stress (Stock et al., 2000). The identification of heat shock-related genes, including *dnaJ*, further supports the ability of these strains to maintain growth at elevated temperatures by stabilizing unfolded proteins (Schumann, 2003; Craig et al., 2021).

Given the increasing prevalence of abiotic stresses driven by climate change, resilient PGP endophytes represent valuable candidates for supporting crop performance in stress-prone environments. The observed tolerance to heat, salinity, and drought, suggests that the evaluated strains may contribute to plant stress resilience by sustaining microbial activity and cellular homeostasis under adverse conditions (Grover et al., 2011). PGP-mediated stress alleviation is primarily attributed to the activation and priming of host antioxidant and stress-responsive pathways that regulate ROS homeostasis. While systemic stress tolerance is largely driven by plant-derived antioxidant and metabolic responses, studies have also demonstrated that exogenous application of compatible solutes and osmoprotectants, such as proline, glycine betaine, and trehalose, can alleviate stress symptoms in plants (Hanif et al., 2021; Avendaño et al., 2025). These findings support a localized role for microbially derived metabolites in modulating stress at the root-rhizosphere interface. Further investigation of additional stress-related metabolites and regulatory pathways could help elucidate additional adaptive strategies employed by such bacteria and their functional relevance under field conditions.

### 4.3 Evaluation of biocontrol properties in selected strains

Several bacterial strains tested produced biosurfactants, with variation observed both within and between genera. The highest oil dispersion activity was observed in *Bacillus* strains, which is consistent with previous reports identifying *Bacillus* as one of the most efficient microbial biosurfactant producers (Kim et al., 1997; Ghojavand et al., 2008; Rani et al., 2020). Notably, dispersion capacity varied among the tested *Bacillus* strains, with some demonstrating performance comparable to the synthetic surfactant Triton X. This variability likely reflects strain specific genetic differences and regulatory control of non-ribosomal peptide synthases, which influence biosurfactant composition, structure, and yield (Ongena and Jacques, 2008).

Functionally, biosurfactants contribute to several interconnected biological processes that enhance bacterial competitiveness and biocontrol performance. By reducing surface tension, they increase the bioavailability of hydrophobic, water-insoluble nutrients, thereby improving nutrient acquisition (Shreve et al., 1995). In addition, biosurfactants modify bacterial cell surface hydrophobicity, influencing attachment, motility, along with biofilm formation and maturation (Rosenberg, 1993). Beyond physicochemical effects, biosurfactants have also been implicated in quorum sensing modulation, further supporting coordinated community-level responses (Ron and Rosenberg, 2001). Collectively, these functions link biosurfactant production to effective surface colonization, community stability, and competitive fitness.

Importantly, several biosurfactants produced by *Bacillus* species belong to cyclic lipopeptide families that also exhibit antimicrobial properties, thereby directly linking their production to biocontrol potential. In this study, *Bacillus* strains exhibited inhibitory activity against multiple fungal species, with two strains effectively inhibiting *F. oxysporum*, *F. graminearum*, and *R. solani*-AG3. The higher antifungal activity observed in the *Bacillus* strains, correlates with the higher biosurfactant activity observed in the oil dispersion assays. *Bacillus* species are well documented producers of cyclic lipopeptides, such as surfactin, iturin, and fengycin, which have been widely reported for their antifungal activity (Islam et al., 2012; Fan et al., 2017; Xiao et al., 2021). Moreover, individual *Bacillus* strains are capable of co-producing multiple biocontrol compounds, which may explain the broad-spectrum inhibition observed among the top-performing strains (Athukorala et al., 2009; Kim et al., 2010).

WGS revealed that the top inhibitory strains harbored multiple BGCs associated with compounds with known antifungal activity (**Table 5**). Fengycin and plipastatin are known to inhibit a broad range of filamentous fungi by disrupting cell membrane integrity, leading to cell death (Vanittanakom et al., 1986; Fazle Rabbee and Baek, 2020). Bacilysin inhibits fungal growth through the release of anticapsin, which targets glucosamine-6-phosphate synthase, thereby impairing cell wall biosynthesis (Wang et al., 2018). In addition, bacillibactin contributes indirectly to antifungal activity through iron sequestration, limiting its availability to competing fungal pathogens (Dimopoulou et al., 2021). While surfactin alone does not exhibit strong antifungal activity, it has been reported to act synergistically with fengycin, amplifying overall biocontrol efficacy and supporting important functions such as improved swarming motility, surface colonization, and biofilm formation (Kim et al., 2010; Zhang et al., 2020). In combination with resistance-associated genes such as *aadK* and *mph(K)*, these traits likely improve bacterial persistence and competitiveness for space and nutrients (Luo et al., 2015). Although, not directly evaluated in this study, *Bacillus* species are also known to produce a range of diffusible, non-volatile antifungal compounds, which may further contribute to their effectiveness (Zhang et al., 2020).

This study characterized culturable endophytic *Bacillus* and *Pseudomonas* strains isolated from *C. sativa* and *C. majus* through integrated genomic and functional analyses to evaluate their crop biostimulant and biocontrol potential. The examined strains exhibited diverse plant growth-promoting traits, including indole production, nutrient solubilization, siderophore production, biosurfactant synthesis, and biofilm formation, collectively associated with effective root colonization, competitive exclusion of pathogens, and environmental resilience. Abiotic stress tolerance assays, supported by genomic identification of stress-related genes, demonstrated functional resilience under heat, salinity, and drought-related conditions. Furthermore, selected *Bacillus* strains exhibited strong antifungal activity and biosurfactant production consistent with the presence of multiple BGCs associated with cyclic lipopeptides and antimicrobial compounds.

The pronounced strain-level variability observed across plant growth-promoting, stress tolerance, and antifungal traits highlights the necessity of strain-specific screening and selection when developing microbial formulations for agricultural use. Collectively, these findings highlight the multifunctional potential of the endophytes identified in this study and support their relevance for sustainable crop production, particularly in stress-prone environments. Future work should validate their performance under greenhouse and field conditions to assess their effectiveness in agriculturally relevant settings. Integrating plant phenotypic and molecular analyses will further elucidate the underlying mechanistic pathways. These evaluations will be essential to determine their consistency, scalability, and translational potential.

## Data Availability

The original contributions presented in the study are publicly available. This data can be found here: NCBI BioProject PRJNA1379652.
