# Supplementary material for: Characterization of bacterial endophytes isolated from Cannabis sativa L. and Chelidonium majus L. for their application as biostimulants and biocontrol agents
Source: Front Microbiomes. 2026 Jun 8;5:1780965. doi: 10.3389/frmbi.2026.1780965 (PMC13284072; doi:10.3389/frmbi.2026.1780965)
Supplement: Supplementary file 1 [file DataSheet1.docx]

**Supplementary Material**


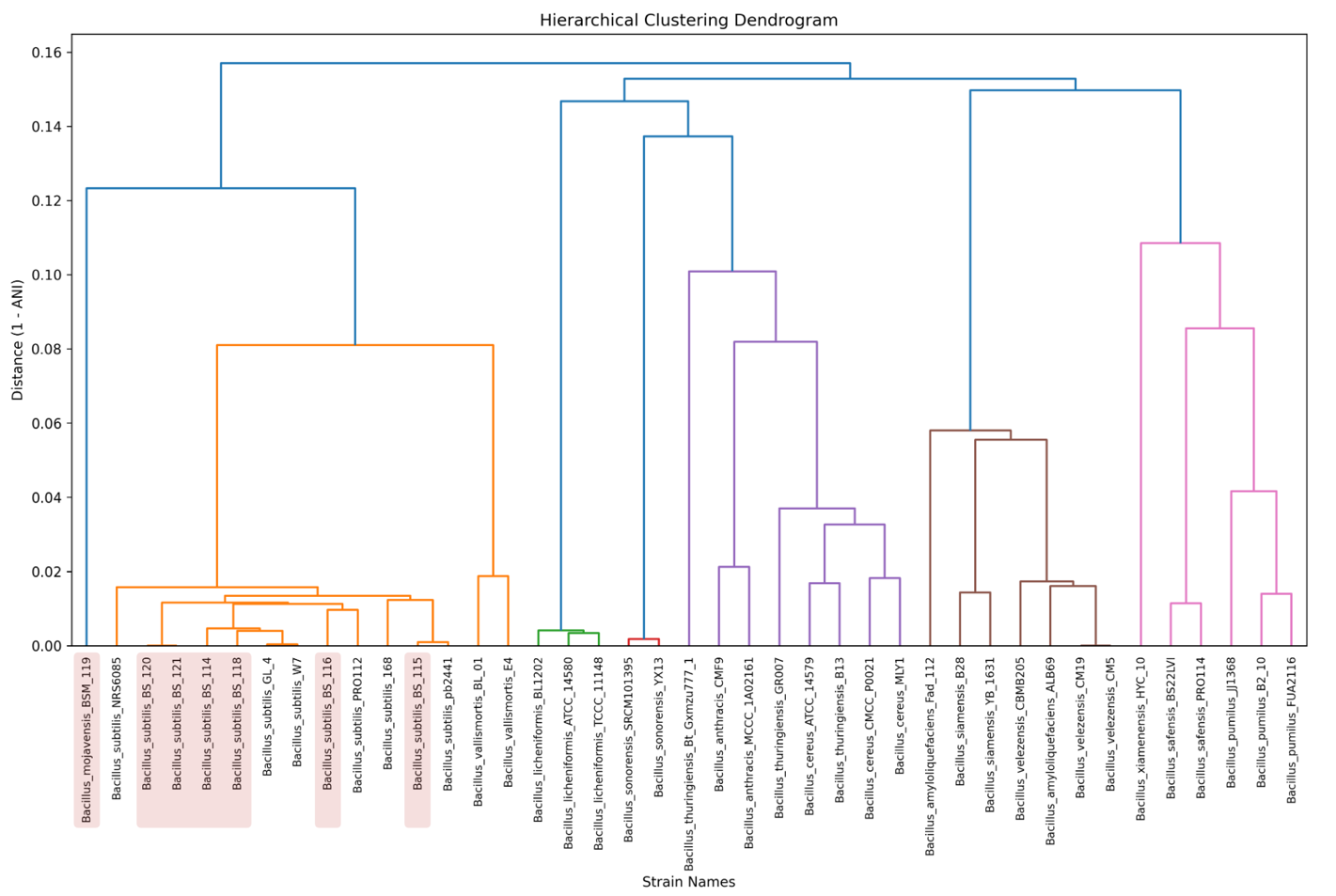


Supplementary Figure 1. Hierarchical dendrograms of selected *bacillus* strains based on distance matrix to compare relations


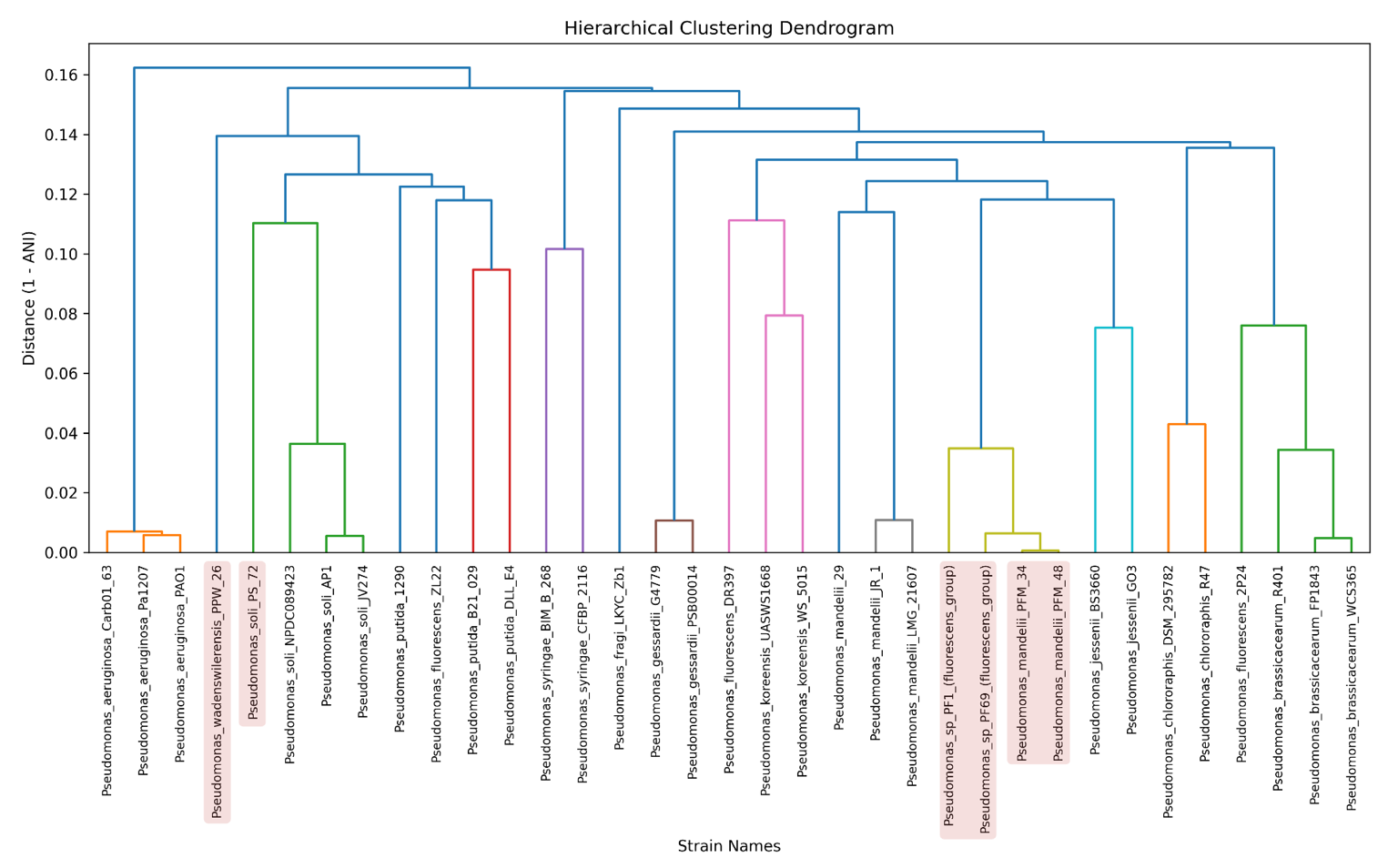


Supplementary Figure 2. Hierarchical dendrograms of selected *pseudomonas* strains based on distance matrix to compare relations


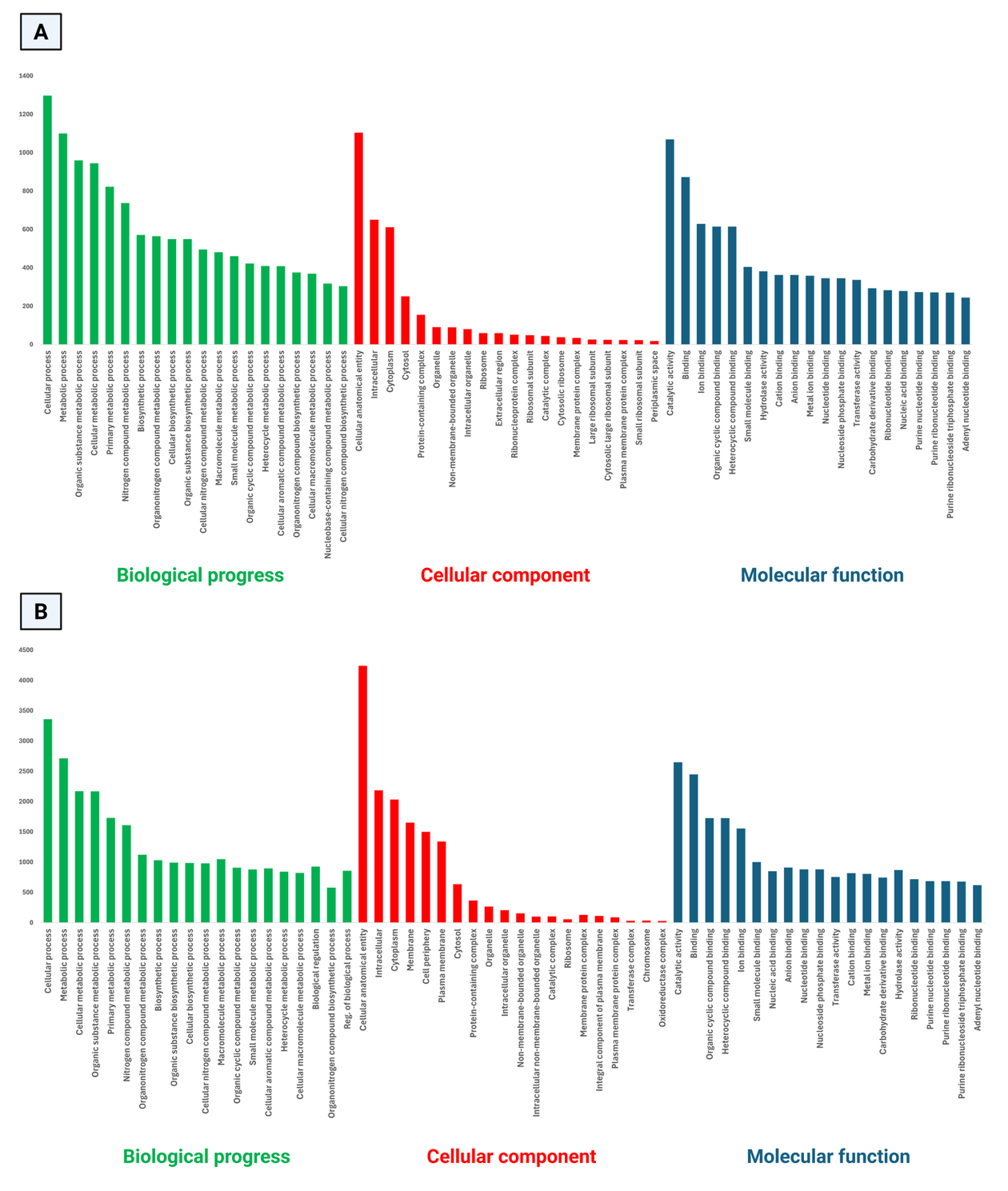


Supplementary Figure 3: Gene Ontology functional classification results of strains (A) BS-116 and (B) PPW-26, showing gene distribution across Biological Processes, Cellular Components, and Molecular Functions.


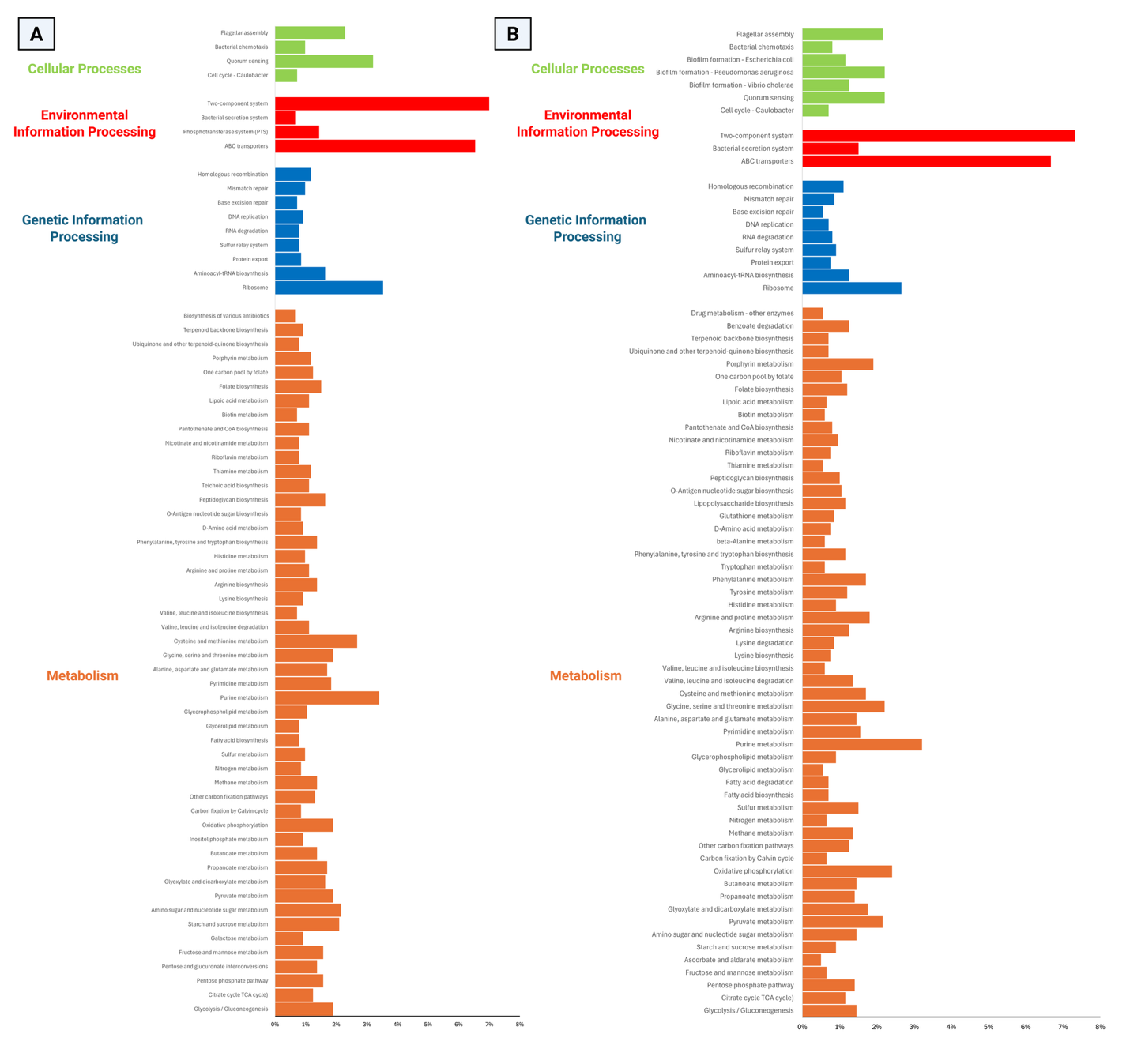


Supplementary Figure 4: KEGG pathway classiﬁcation map of top differentially expressed genes of strains (A) BS-116 and (B) PPW-26
